# Novel HDAC5-interacting motifs of Tbx3 are essential for the suppression of E-cadherin expression and for the promotion of metastasis in hepatocellular carcinoma

**DOI:** 10.1038/s41392-018-0025-6

**Published:** 2018-08-24

**Authors:** Liang Dong, Qi Dong, Ying Chen, Yichen Li, Bao Zhang, Fanghang Zhou, Xiaoming Lyu, George G. Chen, Paul Lai, Hsiang-fu Kung, Ming-Liang He

**Affiliations:** 1 Department of Biomedical Sciences, City University of Hong Kong, Hong Kong, China; 2 School of Public Health and Tropical Medicine, Southern Medical University, 1023 Shatai Road, 510515 Guangzhou, China; 3 Department of Surgery, Faculty of Medicine, The Chinese University of Hong Kong, Hong Kong, China; 4 Key Laboratory of Tumor Immunopathology, Ministry of Education of China, and Institute of Pathology and Southwest Cancer Center, Southwest Hospital, Third Military Medical University, 400038 Chongqing, China; 5 Biotechnology and Health Center, CityU Shenzhen Research Institute, Shenzhen, China

## Abstract

Tbx3, a transcriptional repressor, is essential in the organogenesis of vertebrates, stem cell self-renewal and differentiation, and the carcinogenesis of multiple tumor types. However, the mechanism by which Tbx3 participates in the metastasis of hepatocellular carcinoma (HCC) remains largely unknown. In this study, we show that Tbx3 was dramatically upregulated in clinical HCC samples and that elevated expression of Tbx3 promoted cancer progression. To determine the underlying mechanism, systematic glycine scan mutagenesis and deletion assays were performed. We identified two critical motifs, ^585^LFSYPYT^591^ and ^604^HRH^606^, that contribute to the repression of transcriptional activity. These motifs are also essential for Tbx3 to promote cell migration and metastasis both in vitro and in vivo via the suppression of E-cadherin expression. More importantly, Tbx3 directly interacts with HDAC5 via these motifs, and an HDAC inhibitor blocks Tbx3-mediated cell migration and the downregulation of E-cadherin in HCC. As Tbx3 is involved in the carcinogenesis of multiple types of human cancers, our findings suggest an important target for anti-cancer drug development.

## Cancer: Protein interaction underpins metastasis of liver tumors

A regulatory protein that represses gene activity interacts with an enzyme involved in chromosome remodeling to promote the migration and metastasis of liver cancer cells. Ming-Liang He from the City University of Hong Kong and colleagues found that levels of the T-box transcription factor Tbx3 were dramatically increased in tissue biopsies of liver tumors. They injected Tbx3-expressing human liver cancer cells into mice and saw a positive correlation between Tbx3 activity and cancer progression. By mutating and deleting parts of Tbx3, the researchers identified two particular stretches of the protein that bind histone deacetylase 5, an enzyme involved in ensuring DNA coils, are wound tight to suppress gene activity. This interaction is needed for Tbx3’s tumor-promoting function and may be targetable with drugs in order to prevent metastasis in patients with aggressive liver cancer.

## Introduction

Hepatocellular carcinoma (HCC), which is the third most common malignancy worldwide, is particularly common in the eastern and southeastern Asia and Africa.^1^ Approximately 800,000 new HCC cases are diagnosed and 750,000 deaths are reported annually.^2–4^ Among them, near 50% of new and fatal cases occur in China,^5^ and the overall 5-year survival rate is quite low.^6,7^ However, the underlying mechanisms of hepatocarcinogenesis are not yet well understood.

The T-box (Tbx) family of transcription factors is known to play an essential role in vertebrate development and is characterized by a highly conserved DNA-binding domain, the T-box domain.^8^ Tbx3, a member of the Tbx family, has attracted particular attention since haploinsufficiency or mutation of the Tbx3 gene causes human Ulnar-mammary syndrome (UMS).^9,10^ UMS is a disease that affects the ulnar ray of the limb from the terminal phalanx and that may result in hypoplasia of the fifth digit to complete absence of the forearm and hand.^11^ Patients with UMS exhibit abnormal development of the breasts, teeth, and genitalia.^9^ Moreover, Tbx3 is a signature factor that participates in the specification of the posterior limb mesoderm and in the establishment of the dorsal/ventral limb axis.^12^ During organogenesis, Tbx3 plays pivotal roles in the formation of the heart, liver, and retina.^12–18^ It has been shown that Tbx3 shares a highly conserved DNA-binding domain and repression domain (RD) with Tbx2.^19–21^ The fundamental importance of both Tbx3 and Tbx2 has been demonstrated in the self-renewal process of stem cells, in improvements in the germ-line competency of induced pluripotent stem cells (iPS), cellular senescence and oncogenesis of various cancers including head and neck squamous cell carcinoma, gastric, breast, cervical, bladder and liver cancers, as well as melanoma.^22–39^ Recently, Tbx2 and Tbx3 were also found to be critical mediators of drug resistance in cancer cells.^35,40,41^

Tbx3 is a transcriptional repressor, and its transcriptional RD bears an essential role in suppressing the downstream targets.^42^ Our previous bioinformatics analyses revealed a unique RD that does not share any conserved motif or element with any other known transcriptional repressors.^35^ A detailed analysis of such a domain would not only help us further understand the molecular mechanisms of transcriptional repression mediated by Tbx3 but also reveal the underlying basis of oncogenesis.

Ectopic expression of Tbx3 has suggested that this protein has the ability to increase the invasiveness of melanoma cell lines.^43^ In this study, we provide evidence that two small motifs contribute to transcriptional repression and serve as the interacting sites for HDAC5 in the suppression of cell invasion/migration and metastasis via the deregulation of E-cadherin expression. Our results provide in-depth information for the manipulation of stem cell self-renewal/differentiation and anti-cancer drug development.

## Results

### Elevated Tbx3 protein in liver cancer tissues and the promotion of hepatoma progression

**Fig. 1:**
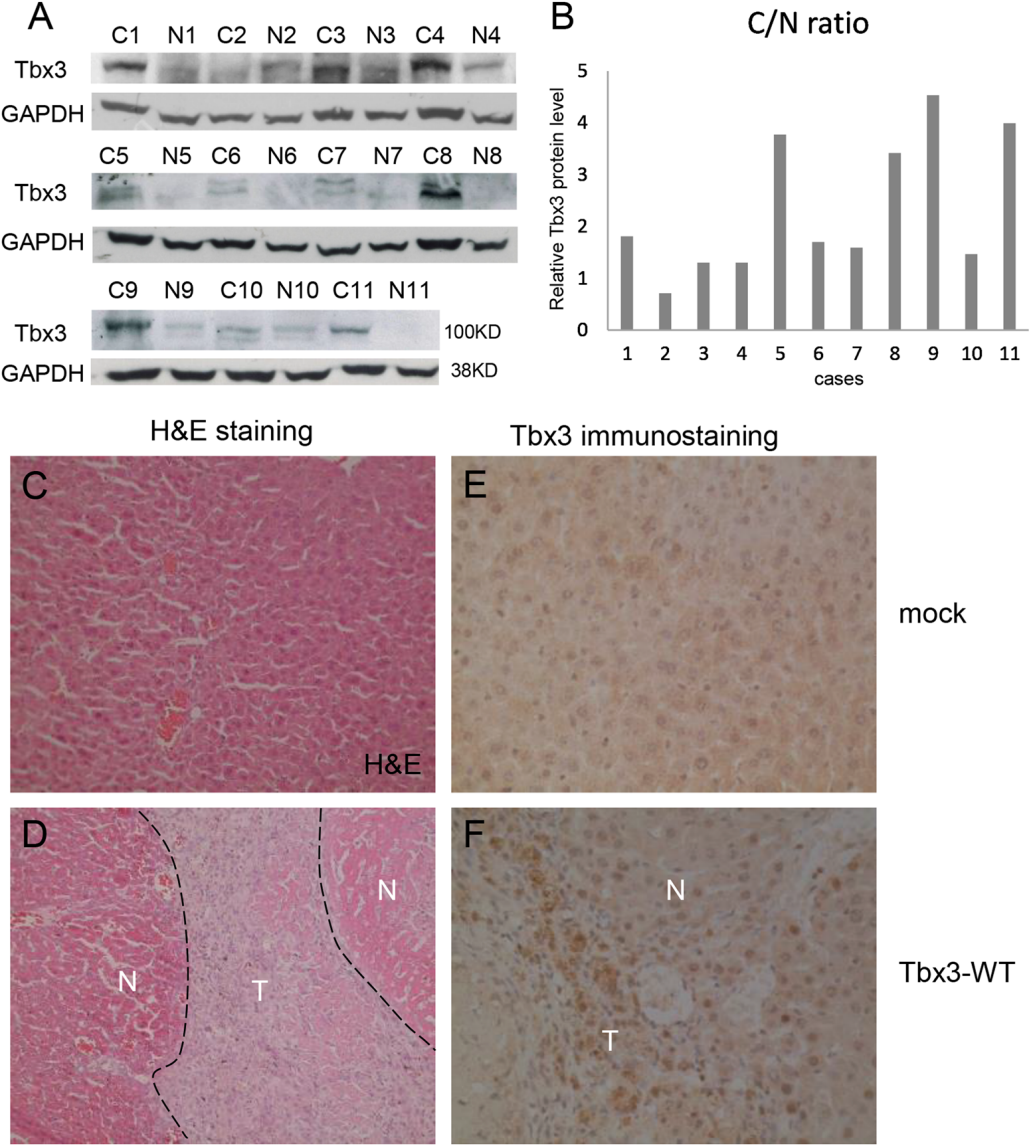
Tbx3 is highly expressed in hepatocarcinoma tissues and promotes tumor progression. **a** A high level of Tbx3 protein was detected in HCC tissues (C) compared with adjacent non-tumor tissues (N) by western blot assays. **b** The ratios of Tbx3 protein levels between clinical HCC tissues (C) and adjacent non-tumor tissues (N) were quantified; a C/N ratio > 1 indicates that the Tbx3 protein level was upregulated in the corresponding HCC patient tissue. **c**, **d** Large tumors were observed when HepG2 cells with ectopic expression of Tbx3 were inoculated into the livers of nude mice via tail vein injection (D) compared with the control (C) Hematoxylin & eosin (H&E) staining. **e**, **f** Immunohistochemical staining shows that Tbx3 was highly expressed in the tumor (D) but not in normal liver tissues (C)

To reveal the expression status of Tbx3 in HCC, we detected the level of Tbx3 protein in 11 paired HCC tumors and adjacent non-tumor tissues by western blot assays. As shown in Fig. 1a, the levels of Tbx3 protein in 8 of the 11 pairs of HCC tissues were dramatically upregulated as compared with the non-tumor tissues. The ratios of the Tbx3 protein levels between tumors and non-tumor tissues were scanned and quantified (Fig. 1b). To determine the role of upregulated Tbx3 in hepatocarcinogenesis, Tbx3 was stably transfected into HepG2 cells, which were then inoculated into nude mice through tail vein injection. Then, we isolated the tumors and performed hematoxylin and eosin (H&E) staining and immunohistochemistry (IHC). We showed that elevated Tbx3 expression was positively correlated with HCC progression (Fig. 1c–f). Taken together, these data suggest that a high Tbx3 level promotes tumor progression.

To further confirm the effect of Tbx3 in HCC development, Tbx3-specific siRNA was used for loss-of-function assays in HCC cell lines (HepG2 and Bel7404 cells). RT-qPCR analysis revealed that Tbx3 was obviously downregulated in both HepG2 and Bel7404 cells as compared with the NC (Fig. S2). Wound healing assays revealed that the knockdown of Tbx3 clearly decreased the migration ability of HepG2 and Bel7404 cells (Fig. S1).

### The highly conserved RD in vertebrate Tbx3

An RD of Tbx3 was identified between the Met^541^ and Asp^651^ residues (hTbx3, reference sequence NM_005996) or the Met^558^ and Asp^647^ residues (xTbx3, reference sequence NM_001085611).^23^ This RD is highly conserved between Tbx2 and Tbx3 in vertebrates.^19,35^ Other studies have revealed that such an RD is essential for Tbx2/3-mediated cell senescence and tumorigenesis.^19,44^ In our previous study, a unique RD was found only in Tbx2 and Tbx3 through a genome-wide search. The RD is composed of ~90–110 residues among different species and does not share any conserved sequence or motif with other known RDs.^45^ After the sequences of Tbx2 and Tbx3 were aligned and compared among different vertebrates (i.e., from Zebrafish to human), only 56 residues were found to be highly conserved between Leu^558^ and Ser^623^ with 80% sequence identity.^45^ *Xenopus* Tbx3 is the shortest and most conserved sequence, and it lacks the additional variable residues between Ala^596^ and Arg^614^. Apart from the additional variable residues, *Xenopus* Tbx3 shares 54 (*X.l*, 54/56, 96% identity) or 55 (*X.t*, 55/56, 98% identity) identical residues with human Tbx3. We postulated that key motif(s) might play a pivotal role in transcriptional repression in this highly conserved region.

### Identification of repression motifs by combined mutations

**Fig. 2:**
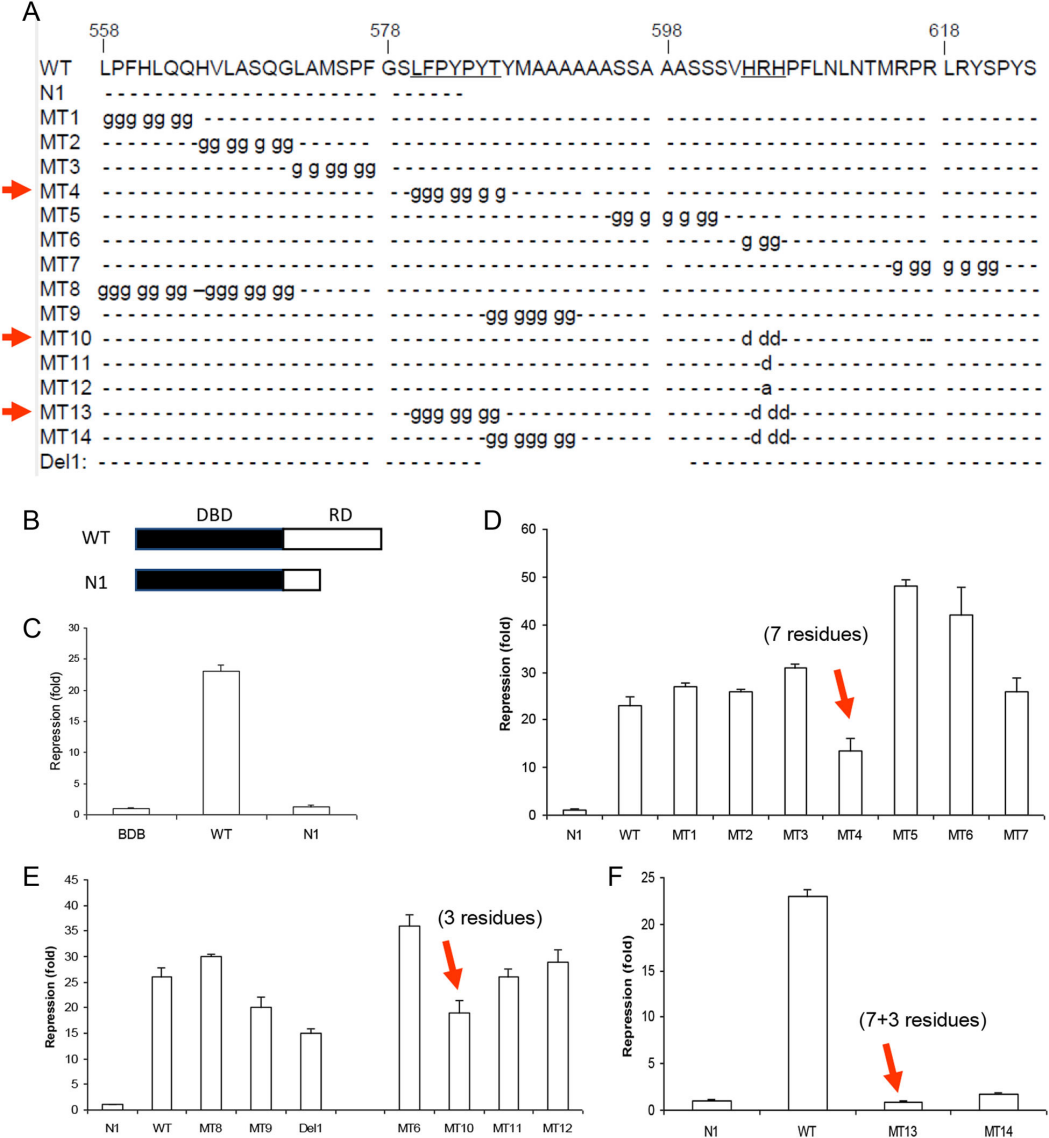
Identification of repression motifs by combination mutations. **a** Diagram of deletion and residue substitution scan mutations in the Tbx3 repression domain. WT wild type, N1 the N-terminal portion of the RD, MT mutant, Del internal deletion. **b** Diagram of the Gal4-RD fusion protein. **c** The transcriptional repression activity of the wild-type RD and the conserved N-terminal half of the RD that fused with the Gal4 DNA-binding domain (DBD). **d** The transcription repression activity of glycine scan mutations. **e** The transcriptional repression activity of an individual “motif”. **f** The transcriptional repression activity of two important motifs combined. The mean value of the N1 control was defined as 1. The values of three independent experiments are expressed as the mean ± standard deviation

The C-terminal half sequence (after Tyr^588^) was truncated to examine the function of the highly conserved N-terminal half sequence of the RD (Fig. 2a). As shown in Fig. 2b, c, the luciferase expression was reduced by 25-fold when wild-type RD was fused with the Gal4 DNA-binding domain (BDB). However, the truncated N-terminal half of the RD (Met^558^~Try^588^) failed to demonstrate any repression activity, which suggests that the C-terminal half is crucial for maintaining normal transcriptional repression activity. This C-terminal truncated sequence was employed as a negative control for the remaining studies.

In order to identify potential crucial motif(s) with transcriptional repression activity, a glycine scan mutation assay was performed. As shown in Fig. 2a, the conserved residues were mutated into stretches of glycines (MT1–MT7), and the corresponding effect was reflected by the luciferase activity. Substitution of ^563^LPFHLQQ^569^ (MT1), ^571^VLASQGL^577^ (MT2), ^579^MSPFGG^584^ (MT3, Gly replaced with Ala), and ^613^RPRLRYN^619^ (MT7) with glycine had no effect on transcriptional repression activity. Compared with the wild-type control, the repression activity was only reduced by 40% (24-fold vs 16-fold) when ^585^LFSYPYT^591^ was substituted with glycine (MT4). We then induced a combined mutation by substituting all the residues of ^566^LPFHLQQ^569^ and ^571^VLASQGL^577^ with glycine (MT8) and then examined the repression activity. As shown in Fig. 2e, no significant change was observed.

It is possible that the flanking sequences ^593^MAAAAAA^599^ and ^604^HRH^606^ at the C-terminus might be critical. Generation of a combined mutation was attempted in order to substitute the ^585^LFSYPYTYMAAAAAA^599^ residues with glycine. However, the generation of such a mutant was unsuccessful because the GC-content was too high in the primer binding site. Therefore, an internal deletion was generated to remove all these residues (Del1), and the repression activity was found to be reduced by 42%, which was similar to the effect of substituting ^585^LFSYPYT^591^ with glycine. To further reveal the effect of flanking residues ^592^YMA^594^ at the C-terminus of ^585^LFSYPYT^591^, we substituted ^588^YPYTYMA^594^ with glycine (MT9), but repression was only reduced by 25%.

The ^604^HRH^606^ are three positively charged residues within the conserved sequence in Tbx3. We observed that HRH can be replaced by SRS, PRN, or SRN in Tbx2 in different vertebrates. In all cases, the Arg^605^ residue has never been replaced by other amino acids. We postulated that Arg^605^ might be very important in the context of the RD. We, therefore, substituted Arg^605^ along with alanine (MT12) and aspartic acid (MT11). The number of positive charges was reduced from 3 to 2 (MT12) and 1 (MT11, same as Tbx2), respectively. No obvious change in repression activity was observed (Fig. 2e). However, when all three positively charged residues were replaced with aspartic acid (charges from +3 to −3, MT10), the repression activity was clearly reduced by 27%.

Based on the observation above, we postulated that the residues ^585^LFSYPYT^591^ and ^604^HRH^606^ in Tbx3, which are located at each ends of an alanine stretch, are required for the transcriptional repression activity through cooperation. To demonstrate our hypothesis, both ^585^LFSYPYT^591^/^604^HRH^606^ (MT12) and ^588^YPYTYMA^594^/^604^HRH^606^ (MT13) were mutated into Gly/Asp_._ As shown in Fig. 2f, the transcriptional repression activity was completely abolished when ^585^LFSYPYT^591^ and ^604^HRH^606^ were simultaneously mutated, yet in the case of the ^588^YPYTYMA^594^/^604^HRH^606^ mutation (MT14), the activity was reduced by 93%, which further demonstrates the importance of the ^588^YPYT^591^ residues.

### Novel motifs are essential for the promotion of HCC cell migration and metastasis by Tbx3

**Fig. 3:**
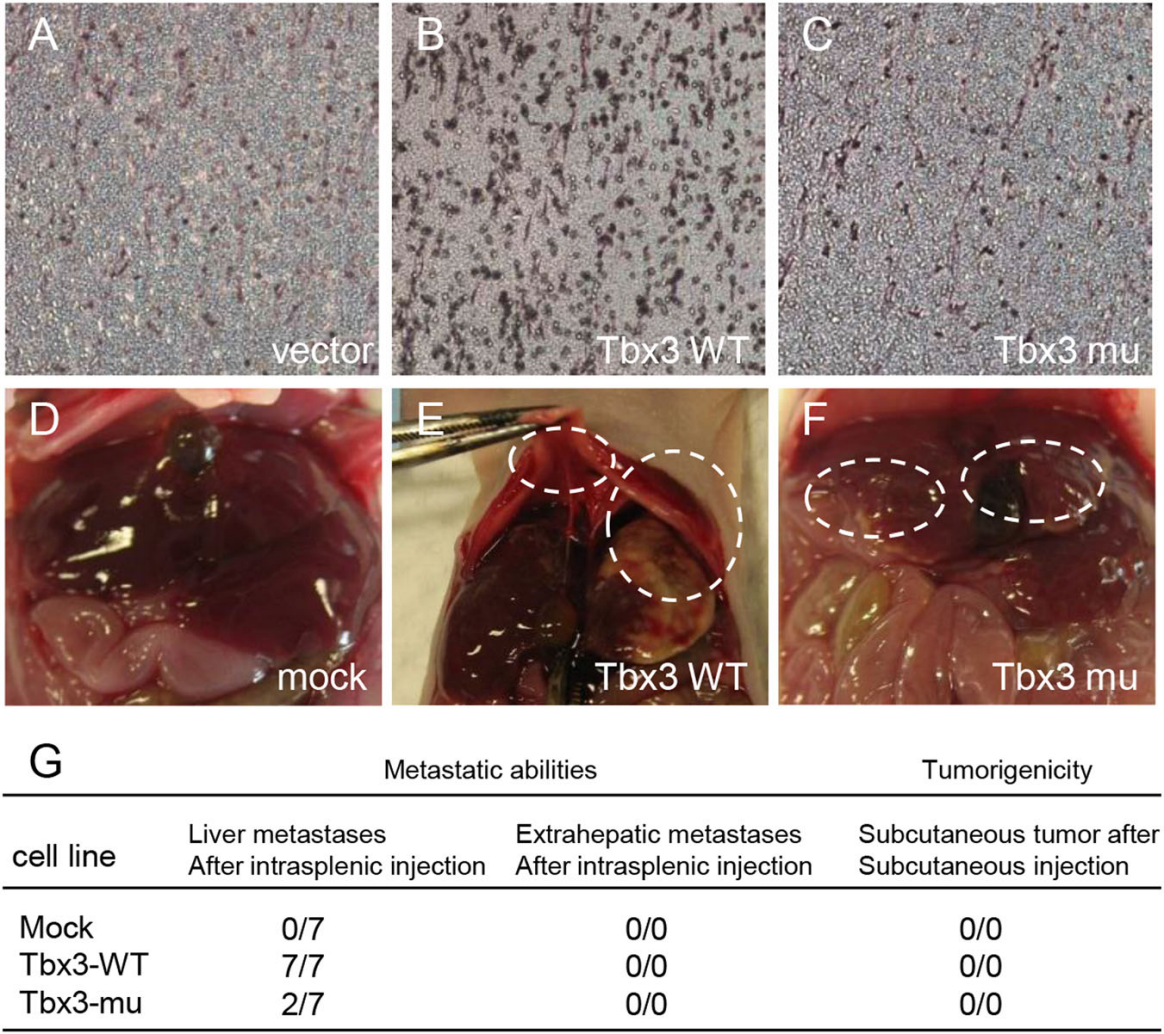
Novel repression motifs are essential for the regulation of HCC cell metastasis by Tbx3. **a**–**c** Transwell assay of cell invasion in stable HepG2 cells that overexpress Tbx3 WT or Tbx3 mutant. **d**–**f** Overexpression of Tbx3 WT in HepG2 cells induced liver metastasis more frequently than the cells with expression of the Tbx3 mutant after intrasplenic injection. **g** Metastatic ability and tumorigenicity of HepG2 cells with wild-type Tbx3 or mutant Tbx3 in nude mice

As the ^585^LFSYPYT^591^ and ^604^HRH^606^ motifs were required for Tbx3 to repress transcription, we postulated a crucial role of the novel motifs in HCC metastasis. We simultaneously mutated the ^585^LFSYPYT^591^ and ^604^HRH^606^ motifs into glycine and performed a Transwell assay to determine their effects on cancer cell invasiveness. We showed that the ectopic expression of Tbx3 was able to upregulate HepG2 cell migration, while the Tbx3 mutant did not differ from the control group (Fig. 3a–c). Similar results were also obtained in Bel7404 hepatoma cells (Fig. S4A-S4C). Cancer metastasis assays were also used for in vivo experiments in nude mice. HepG2 cells with stable ectopic expression of Tbx3 or Tbx3 mutant were implanted in the spleen, and tumor modules that developed in the liver were examined at the end of the experimental period. Consistently, ectopic expression of Tbx3 largely promoted tumor metastasis. Large tumor modules were observed in all the test mice that received implanted cells that ectopically expressed wild-type Tbx3 (7 out of 7 mice, Fig. 3e), while no tumor nodules were observed in the control group (Fig. 3d). Interestingly, implantation of cells that expressed the Tbx3 mutant almost completely abolished the metastatic effect (Fig. 3f). Our data reveal that the ^585^LFSYPYT^591^ and ^604^HRH^606^ motifs of Tbx3 play crucial roles in the regulation of HCC cell migration and metastasis.

### Repression motifs are required for the suppression of E-cadherin expression by Tbx3

**Fig. 4:**
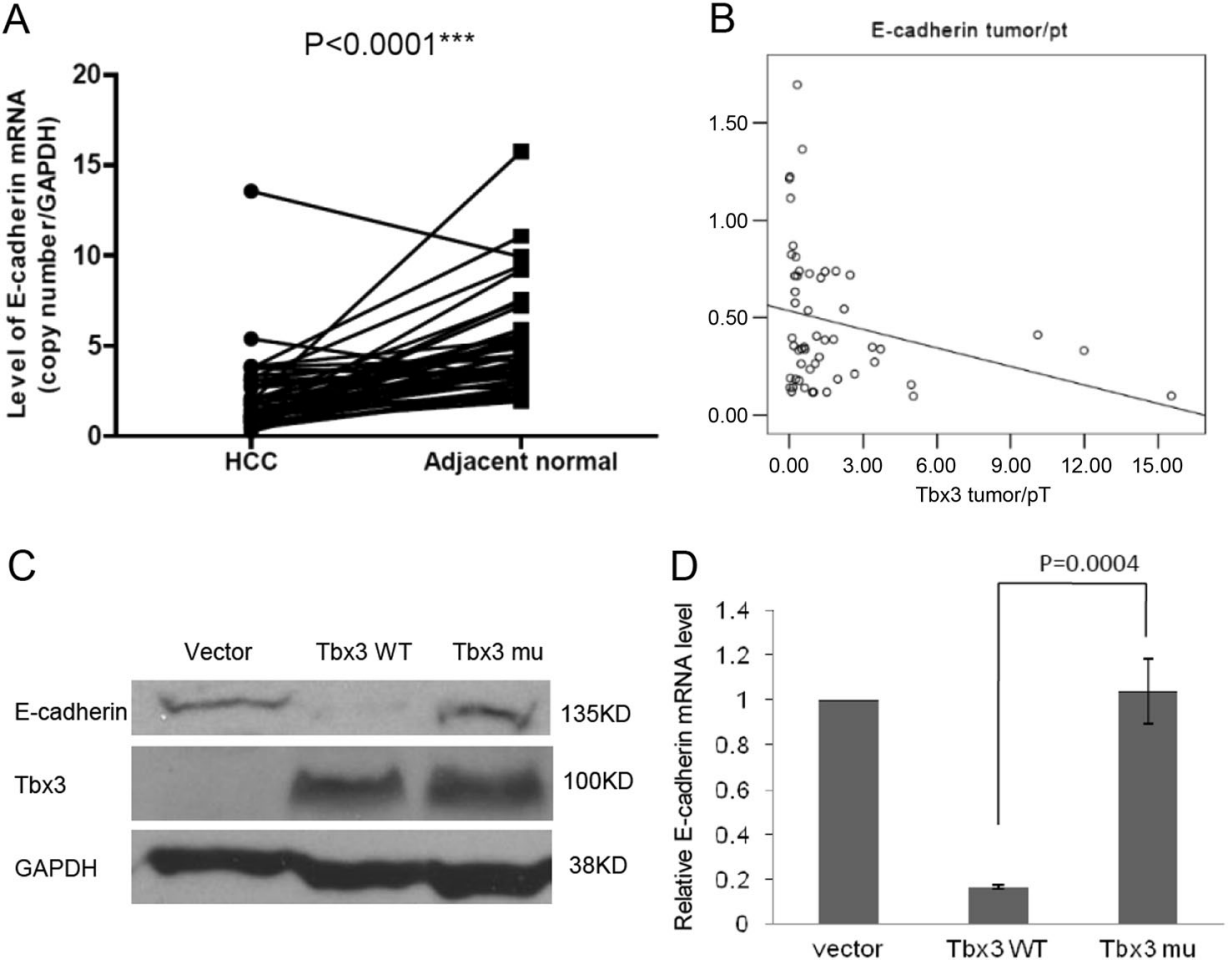
Repression motifs are required for the regulation of E-cadherin expression by Tbx3. **a** A low level of E-cadherin expression was found in 53 paired primary HCC tissues compared with adjacent non-tumor tissues by RT-qPCR. **b** Negative correlations were observed between Tbx3 and E-cadherin expression in clinical HCC samples subjected to RT-qPCR; statistical analysis was performed using SPSS software. **c**, **d** According to WB (C) and RT-qPCR (D) assays, Tbx3 repressed E-cadherin expression whereas the Tbx3 mutant had no obvious effect on E-cadherin expression in HepG2 cells

We quantified the copy numbers of Tbx3 and E-cadherin mRNA in 53 primary liver cancer tissues (HCCs) and their corresponding adjacent non-tumor tissues by RT-qPCR. The copy number of Tbx3, E-cadherin, and GAPDH mRNA ranged from 87.4 to 66,911, 5130.7 to 261,286.8, and 1273.5 to 238,080, respectively. The ratio of the copy numbers of Tbx3 to GAPDH and E-cadherin to GAPDH represented the standardized Tbx3 and E-cadherin for each sample. The Tbx3 and E-cadherin mRNA expression ratios in the tumor (T) and non-tumor (N) tissues were calculated. R values > 100 indicated that the gene was overexpressed in that case, whereas R values < 100 indicated that the gene was not overexpressed. We observed that 50 of 53 HCCs (94.3%) exhibited a reduced E-cadherin expression pattern as compared with adjacent non-tumor tissues (*p* < 0.0001) (Fig. 4a), which suggests a downregulation of E-cadherin expression in HCC.

To further investigate the correlations between Tbx3 and E-cadherin, we observed that the ratio of the quantity of Tbx3 mRNA between tumor and non-tumor tissues was negatively associated with that of E-cadherin (*R* = −0.303, *p* < 0.05) (Fig. 4b), which demonstrates an obvious reverse-correlation between the expression levels of Tbx3 and E-cadherin.

To verify the functional relationship between Tbx3 and E-cadherin, we depleted endogenous Tbx3 by specific siRNA. We demonstrated that the level of E-cadherin expression was markedly elevated by the knockdown of Tbx3 both in HCC cell lines (HepG2 and Bel7404) and HEK293 cells (Fig. S2). This shows that the E-cadherin expression level is inversely correlated with the Tbx3 expression level in different cell lines. In terms of other EMT-related proteins tested in this study, we observed that the protein levels only displayed minor changes (ZO-1, Vimentin and Claudin) after knockdown of Tbx3 (Fig. S3). These results indicate that Tbx3 may specifically regulate E-cadherin expression to control HCC metastasis. To reveal the effect of the Tbx3 motifs ^85^LFSYPYT^591^ and ^604^HRH^606^ on E-cadherin expression, we further examined the level of E-cadherin protein in HepG2 and Bel7404 cells by western blot. As expected, upon ectopic expression of wild-type Tbx3, the E-cadherin protein expression level was obviously reduced, but the same effect was not observed after expression of the Tbx3 mutant form (Fig. 4c and Fig. S4D). Consistent with the western blot results, we found that, in the setting of wild-type Tbx3 overexpression rather than that of the Tbx3 mutant, the E-cadherin mRNA levels were decreased in HepG2 cells (Fig. 4d). Our results demonstrate that the downregulation of E-cadherin by Tbx3 is primarily dependent on its ^85^LFSYPYT^591^ and ^604^HRH^606^ motifs.

### Tbx3 and HDAC5 cooperate in the regulation of HCC cell migration and E-cadherin expression

**Fig. 5:**
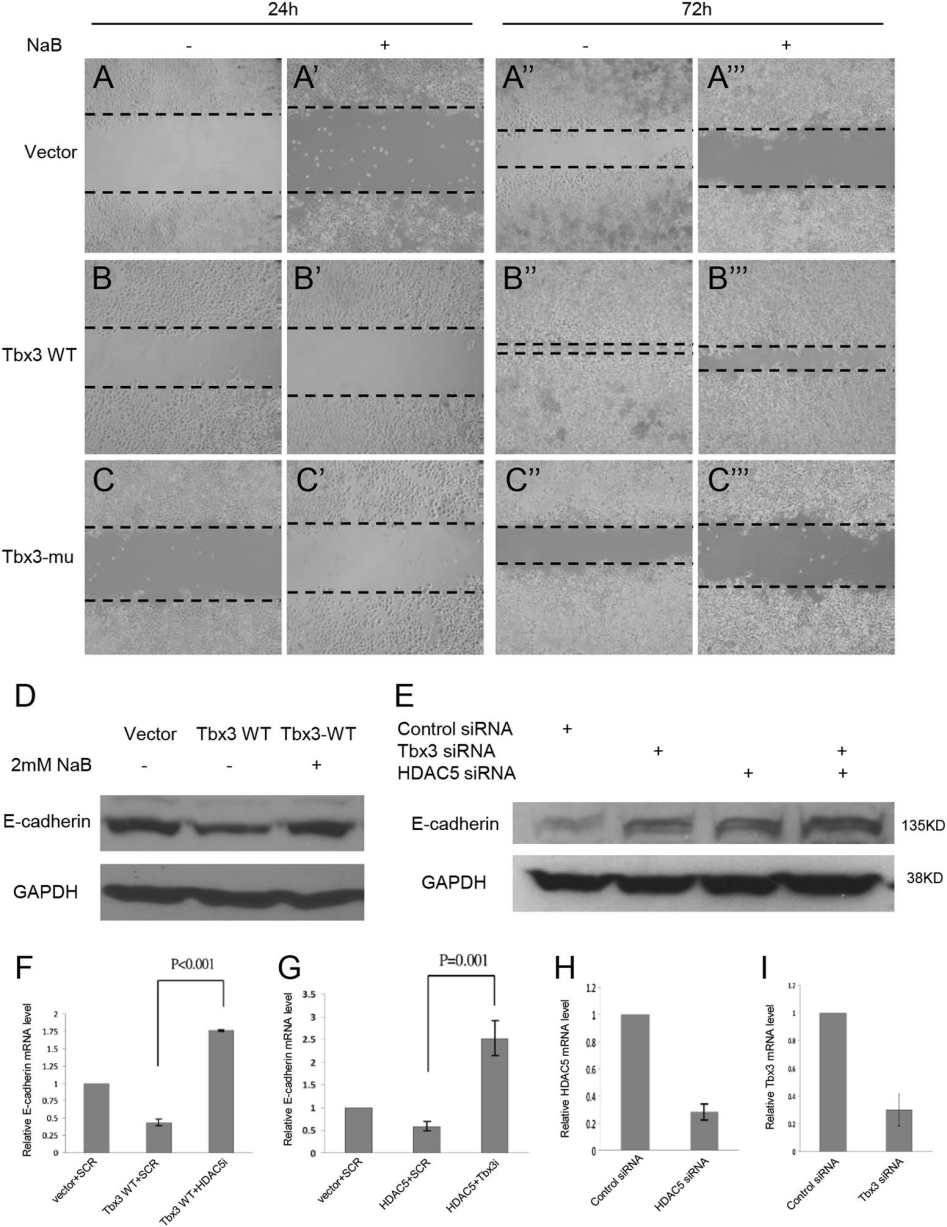
Tbx3 and HDAC5 cooperate in the regulation of HCC cell migration and E-cadherin expression. **a**–**c** Wound healing assay was performed to detect migration of HepG2 cells. Decreased migration of HepG2 cells appeared after treatment with the HDAC inhibitor NaB. **d** WB analysis of E-cadherin in Tbx3 WT or mock cells with or without NaB treatment. **e** WB analysis of E-cadherin in Tbx3 siRNA- or/and HDAC5 siRNA-treated cells. **f**–**i** RT-qPCR analysis of the E-cadherin mRNA level in the indicated transfected cells

To investigate the potential mechanism by which Tbx3 regulates HCC migration, we searched previous studies and found that Tbx3 was able to interact with HDACs.^38^ Therefore, using a wound healing assay, we examined whether treatment with the HDAC inhibitor sodium butyrate (NaB) would affect cell migration in vitro. When sodium butyrate was added to the culture medium, a clear reduction in migration upon either ectopic expression of wild-type Tbx3 or Tbx3 mutant cells was observed (Fig. 5a, a”’). This indicates that, after treatment with an HDAC inhibitor, E-cadherin expression was upregulated, which was required to decrease the cell migration induced by Tbx3 (Fig. 5b, b”’).

To determine whether Tbx3 mediated E-cadherin suppression via HDAC5, we treated the wild-type Tbx3 stable cells with the HDAC inhibitor sodium butyrate. After treatment for 48 h, a recovery in E-cadherin expression was observed (Fig. 5d), which indicates that E-cadherin expression was affected by the HDAC inhibitor. When endogenous HDAC5 or Tbx3 was depleted by siRNAs, HepG2 cells showed a modest increase in the levels of E-cadherin; however, the simultaneous depletion of HDAC5 and Tbx3 significantly increased the protein levels of E-cadherin (Fig. 5e). Moreover, upon knockdown of HDAC5, as shown in Fig. 5f, g, the ectopic expression of either Tbx3 or HDAC5 was able to inhibit E-cadherin mRNA expression, whereas the ectopic expression of one and the concomitant knockdown of the other increased E-cadherin expression. The knockdown efficiency is indicated in Fig 5h, i. Taken together, our data indicate that Tbx3 and HDAC5 function together to regulate HCC cell migration and E-cadherin expression.

### Tbx3 interacts with HDAC5 through the ^585^LFSYPYT^591^ and ^604^HRH^606^ motifs

**Fig. 6:**
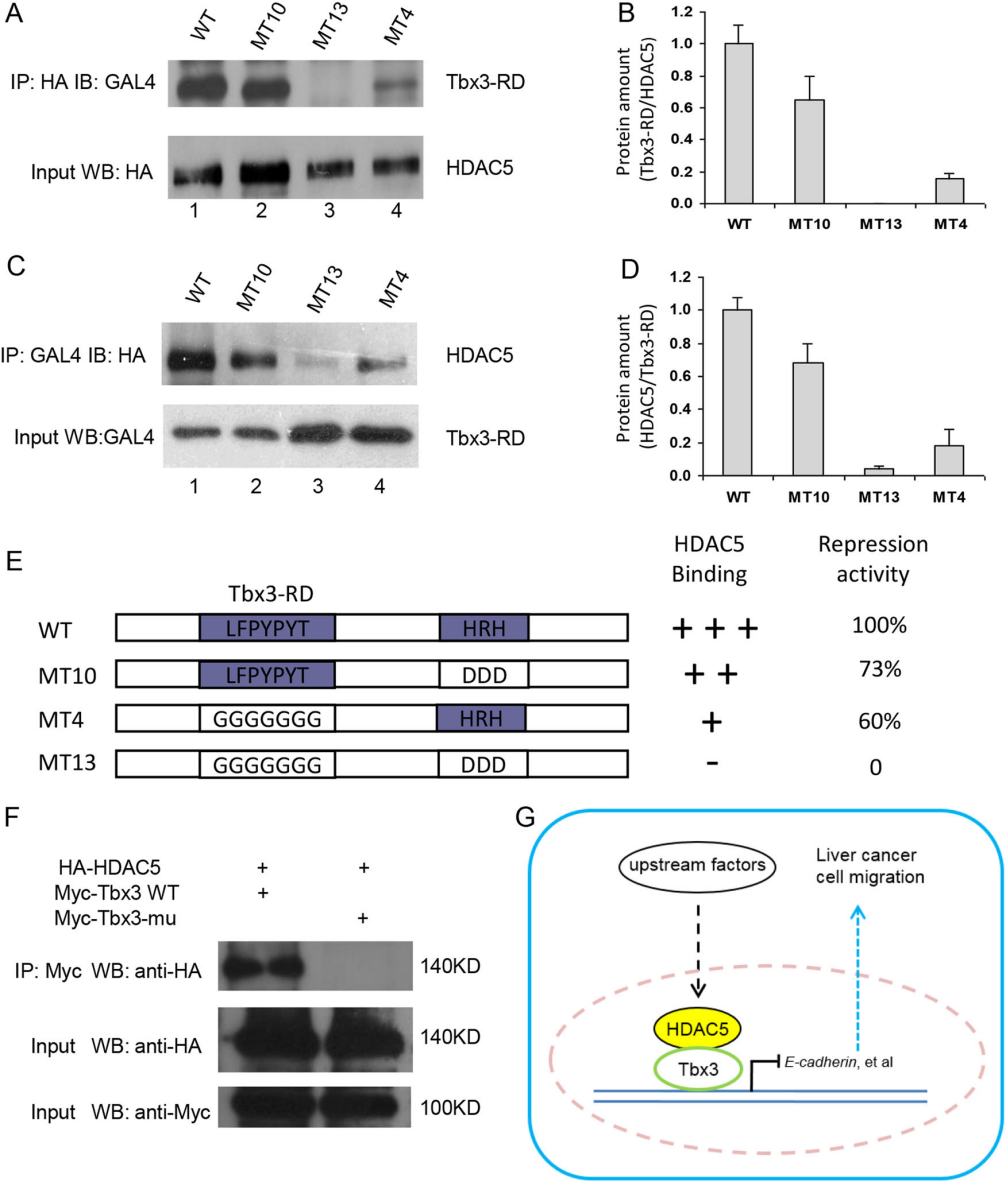
Tbx3 interacts with HDAC5 through the ^585^LFSYPYT^591^ and ^604^HRH^606^ motifs. **a**, **c** An antibody against HA-HDAC5 (**a**) or the DNA-binding domain of Gal4 (**c**) was used to precipitate HDAC5- or RD-binding proteins; the RDs (**a**) or HDAC5 (**c**) were detected by western blot using a specific antibody against the Gal4- or HA-tag (upper panel). The input HA-HDAC5 or RD was detected by a specific antibody against the HA- or Gal4-tag; **b**, **d** The relative protein amounts in A (RD vs HDAC5) and in C (HDAC5 vs RD). The image was scanned, and the density of each band was calculated using Quantity One software. The mean values of three independent experiments are presented with standard deviations; **e** A summary of HDAC5 binding activities and repression activities of the wild type and mutated RD domains of Tbx3; **f** 293 T cells were transfected with Myc-Tbx3 (WT or mutant form) and HA-HDAC5 plasmids. Cell lysate was subjected to IP with the indicated antibody against Myc or HA tag; **g** A proposed working model for Tbx3 and HDAC5 in HCC

To test whether ^585^LFSYPYT^591^ and ^604^HRH^606^ are involved in the interaction of the RD with HDAC5, co-immunoprecipitation and western blot experiments were performed. We showed that the wild-type RD of Tbx3 and HDAC5 would effectively be precipitated by each other (Fig. 6a, c, line 1). The substitution of ^585^LFSYPYT^591^ (MT4) by glycine markedly reduced the binding activity of the RD and HDAC5. When HDAC5 was used as the bait, a weak RD band was observed (Fig. 6a, line 4). Consistently, when the mutated RD was used as the bait, very little HDAC5 was precipitated (Fig. 6c, line 4). After an image density assay, we showed that the binding activity was reduced by ~80% when the ^585^LFSYPYT^591^ residues were replaced with glycine (Fig. 6b, d, e). In the case of ^604^HRH^606^, substitution by aspartic acid mildly reduced the binding activity of RD and HDAC5 (Fig. 6a, c, line 2). Overall, the binding activity was reduced by ~30% in three independent experiments (Fig. 6b, d, e). When both ^585^LFSYPYT^591^ and ^604^HRH^606^ were substituted (MT13), the RD of Tbx3 completely lost its ability to bind to HDAC5 (Fig. 6a, c, line 3). To confirm the results discussed above, we performed a Co-IP assay for Tbx3 and Tbx3 mu (^585^LFSYPYT^591^ and ^604^HRH^606^ mutant form) to verify their interaction with HDAC5. Interestingly, wild-type Tbx3 was able to bind to HDAC5, while the interaction between mutant Tbx3 and HDAC5 was completely abolished (Fig. 6f). Finally, we proposed a working model where Tbx3 uses novel motifs (^585^LFSYPYT^591^ and ^604^HRH^606^) to regulate the metastasis of human HCC through interaction with HDAC5 (Fig. 6g).

## Discussion

Tbx3, a transcriptional repressor, is known to play critical roles in the regulation of multiple cell developmental processes.^46–50^ However, the function and mechanism of Tbx3 in hepatocarcinogenesis is still unclear. In our study, we analyzed the expression of Tbx3 in HCC and paired adjacent non-tumor tissues and discovered that the Tbx3 expression level in the tumor tissues was upregulated compared with that in non-tumor tissues. The ectopic expression of Tbx3 promoted HCC cell migration and metastasis both in vitro and in vivo (Figs. 1 and 5). According to our studies, Tbx3 depletion clearly decreased the migration of HCC cells (Fig. S1). These findings indicated that Tbx3 may contribute to cancer metastasis during the progression of HCC.

Then, systematic mutagenesis assays were performed to identify the key motifs involved in Tbx3 function. Through luciferase reporter assays, we demonstrated that the residues ^585^LFSYPYT^591^ and ^604^HRH^606^ were key motifs that fully contributed to the repression activity of Tbx3 (Fig. 2). The N-terminal deletion actually disrupts the ^585^LFSYPYT^591^ motif and causes complete loss of the ^604^HRH^606^ motif, which explains why a loss of transcription repression activity was observed. Since ^585^LFSYPYT^591^ and ^604^HRH^606^ contributed to the repression activity by 40% and 27%, respectively, and no other repression motif was identified, we postulated that these two motifs might work together. To our surprise, the repression activity was completely abolished when the two motifs were simultaneously mutated. Moreover, both in vitro and in vivo assays demonstrated that they were essential for HCC cell migration and metastasis (Fig. 3). As reported, cancer metastasis is one of the most common prognostic factors. Recent studies have also indicated that Tbx3 may induce melanoma cell invasion via binding the E-cadherin promoter to repress its transcription.^43^ Our results revealed that the repression motifs are required for the regulation of E-cadherin expression by Tbx3 in HCC cells (Fig. 4), which indicates that Tbx3 is a novel modulator of EMT in HCC progression and that it functions through the regulation of E-cadherin expression.

Furthermore, we demonstrated the molecular mechanism of transcriptional repression mediated by the RD of Tbx3. Histone deacetylases (HDACs) catalyze the removal of acetyl groups from histones to condense the chromatin, which leads to transcriptional repression. Due to the lack of a DNA-binding domain, HDACs rely on specific transcription factors to recruit and bind target promoters. In this study, we showed that, by forming a complex with HDAC5, Tbx3 worked together with HDAC5 to regulate E-cadherin expression (Fig. 5) via forming a complex with HDAC5 (Fig. 6). Interestingly, when the transcriptional repression motifs ^585^LFSYPYT^591^ and ^604^HRH^606^ were mutated, their binding activity was greatly decreased, which demonstrates that the binding of ^585^LFSYPYT^591^ and ^604^HRH^606^ to HDAC5 is essential for transcriptional repression. Consistently, the RD did not display any transcriptional repression activity when the binding activity was completely lost by simultaneous mutation of ^585^LFSYPYT^591^ and ^604^HRH^606^. As summarized in Fig. 6, the transcriptional repression activity of RD is highly correlated with HDAC5 binding activity. By contrast, we also assessed the binding of Tbx3 and HDAC1 and found that the interaction between them was very weak and was not obviously affected by mutations in the ^585^LFSYPYT^591^ and ^604^HRH^606^ motifs of Tbx3, and thus the motifs specifically regulate the Tbx3 and HDAC5 complex. These results revealed that the novel HDAC5- interacting motifs of Tbx3 are crucial for the suppression of E-cadherin expression and the promotion of HCC metastasis. Although our Tbx3 mutant has an intact DNA-binding domain, it may still bind to the promoter of E-cadherin; due to the loss of key motifs in the RD that interact with HDAC5, HDAC5 cannot be recruited to the E-cadherin promoter to suppress the transcription, and therefore, it functions in a dominant-negative manner.

In conclusion, we demonstrate that the elevated level of Tbx3 expression is inversely associated with E-cadherin expression in HCC tissues and that high Tbx3 expression indicates a poor prognosis in HCC patients. The ^585^LFSYPYT^591^ and ^604^HRH^606^ motifs play a key role in transcriptional repression of Tbx3 through interaction with HDAC5, which promotes HCC metastasis. Since the ^585^LFSYPYT^591^ and ^604^HRH^606^ motifs are present in Tbx3 and are involved in liver carcinogenesis, our findings may indicate a novel prognostic indicator and an ideal target for anti-cancer drug development.

## Materials and methods

### Reagents

The DNA isolation kit was purchased from Qiagen (Germany). Fetal bovine serum (FBS) was purchased from HyClone Laboratories, Inc. (Logan, UT, USA), while restriction enzymes and DNA ligase were obtained from New England BioLabs Inc. (USA). The dual-luciferase reporter assay system was purchased from Promega (WI, USA), while all other chemicals were obtained from Sigma (St. Louis, MO, USA). The HDAC inhibitor sodium butyrate (NaB) and the selection reagent G418 were purchased from Sigma.

### Clinical tumor specimens

Paired primary HCC specimens were collected from 2004–2009 at Prince Wales Hospital, Faculty of Medicine, The Chinese University of Hong Kong (CUHK). Informed consent was obtained from each patient. This study was approved by the Ethical Committee of Prince Wales Hospital (CUHK).

### Cell culture and luciferase assays

HepG2, BEL7404, and HEK 293 T cells were cultured in Dulbecco’s Modified Eagle’s Medium (DMEM, Invitrogen) supplemented with 10% fetal calf serum at 37 °C in a humidified incubator in an atmosphere of 5% CO2.^51^ HEK 293 T cells in each well of a 12-well plate were transfected with 0.9 μg of test plasmid, 0.1 μg of reporter plasmid, and 5 ng of pRL-CMV (internal control). Transfection was performed with Lipofectamine 2000 (Invitrogen) according to the manufacturer’s instructions. After incubation for 48 h, the cells were harvested and washed once with buffer A (100 mM potassium phosphate, pH 7.0). For luciferase assays, cells from each well were lysed with 150 μl of buffer B (Passive Lysis buffer provided with the Promega Dual-Luciferase Reporter Assay System kit) and then gently agitated for 5 min at room temperature. Luciferase activity was measured from 50 μl of cell lysate using a Luminometer (Promega, WI, USA). Each assay was performed in triplicate and repeated three times. The reporter activity was normalized to Renilla luciferase activity (i.e., the internal control), and the mean value of the negative control was defined as 1.

### DNA constructs and transfection

The luciferase reporter plasmid pJDM1825 was previously described.^19^ This reporter contains five copies of the Gal4 DNA-binding site (CGG AGT ACT GTC CTC CG), which is located upstream of the thymidine kinase promoter that drives the expression of the luciferase gene. A plasmid used to express the fusion protein of Gal4 and the RD of Tbx3 was used to generate deletions and substitution mutants.^19^ To generate the C-terminal deletion of RD, the expression vector was digested with EcoR I and Xba I to remove the cDNA that encodes RD, and then, a PCR fragment coding for 558 M~Y585 (Fig. 2) was used to replace the RD sequence. The N- and C-terminal portions that bear the vector sequences were then amplified by a pair of phosphorylated primers. After self-ligation, an expression vector for the expression of the internal deleted RD was generated. In addition, the substitution mutants were generated using the GeneTailor Site-Directed Mutagenesis System (Invitrogen, CA, USA). The primers used to generate all the constructs are listed in Table S1. All the constructs were confirmed by automated DNA sequencing.

Full-length human Tbx3 cDNA was amplified from DLD-1 cells and cloned into a pIRES-neo vector at BamHI and ClaI sites. The Tbx3 mutant construct was generated using the GeneTailor Site-Directed Mutagenesis System (Invitrogen) according to the manuals and was confirmed by sequencing. WT and mutant Tbx3 cDNA was subcloned into a pcDNA3.1-myc vector for co-immunoprecipitation. The pcDNA3.1-E-cadherin expression plasmid was a gift from Dr. Cara J. Gottardi. HepG2, Bel7404 or 293 T cells were transfected with the pIRES-neo-Tbx3 WT, pIRES-neo-Tbx3 mu, or pIRES-neo plasmids using Lipofectamine 2000. Stable transfections were selected with 600 µg/ml G418, which was added to the medium for 7–10 days, and then, the culture was maintained in 300 µg/ml G418 antibiotic. Stable clones were assessed by RT-PCR and western blot to confirm recombinant protein expression.

Tbx3-, HDAC5-, and E-cadherin-specific siRNAs were designed online (http://www.dharmacon.com/designcenter/DesignCenterPage.aspx) and synthesized by GenePharma. The target sequences are as follows:

Tbx3: 5′-ATGCCAAAGAGGATGTACATTCA-3′,

HDAC5: 5′-AGAAACAGCATGACCACCTGACAA-3′,

E-cadherin: 5′-ACCACAAATCCAGTGAACAACGAT-3′

Cells were seeded into 12-well plates at a density of 5 × 10^4^ cells per well before transfection. siRNA duplex (100 pmol) and a Lipofectamine 2000 (Invitrogen) mixture were transfected into HCC cells according to the manufacturer’s protocol. Then, 48 h later, the cells were harvested and examined for the expression level of various genes by quantitative real-time PCR. The migration capacity of the cells was also examined.

### Western blot

Cells were lysed in RIPA buffer (50 mM Tris-HCl (pH 7.4), 150 mM NaCl, 1% NP-40, 1% deoxycholate, 0.1% SDS, 1 mM EDTA) supplemented with protease inhibitors (Roche) for 30 min on ice and were then centrifuged. The protein concentration was then determined using a Bio-Rad Protein Assay. Western blot experiments were performed as previously described.^52–54^ Briefly, protein (30 μg) was resolved in 10 or 15% polyacrylamide gels and transferred to PVDF membranes (Amersham Pharmacia, USA). The antibodies used for immunoblotting were as follows: mouse anti-GAPDH (sc-51906, Santa Cruz), mouse anti-GAL4 (sc-510, Santa Cruz), rabbit anti-HA (sc-805, Santa Cruz), Myc (Santa Cruz), and E-cadherin (Boster Biological Technology). Horseradish peroxidase-conjugated goat anti-mouse, donkey anti-goat, and goat anti-rabbit antibodies were purchased from Santa Cruz. An ECL detection assay (ECL + ) was performed according to the manufacturer’s protocol. Gel images were acquired with a calibrated GS-800 scanner (Bio-Rad), and the density of the signals was calculated using Quantity One software (Bio-Rad, USA).

### Co-immunoprecipitation

HEK 293 T cells were seeded in 10-cm dishes at 30–50% confluence in DMEM supplemented with 10% FBS. After overnight culture, the cells were co-transfected with an equal amount (10 μg) of expression plasmid encoding Gal4-RDs and HA-HDAC5. Forty-eight hours post-transfection, the cells were harvested and lysed in RIPA buffer (50 mM Tris-HCl (pH 7.4), 150 mM NaCl, 1% NP-40,1 mM EDTA) supplemented with protease inhibitors (Roche) for 30 min on ice. The cell lysate was centrifuged, and protein concentrations were determined using a Bio-Rad Protein Assay. The cell lysate was then incubated with 1 μg of anti-HA (Santa Cruz) or anti-GAL4 (Santa Cruz) antibody overnight at 4 °C. Protein A/G (Santa Cruz, CA) beads were then added, which was followed by an incubation for 2 h at 4 °C. Next, the beads were washed five times in PBS. After centrifugation, the beads were resuspended in SDS-PAGE buffer, heated to 100 °C, and analyzed by western blotting.^55^

### RT-PCR and quantitative real-time PCR

Total RNA was isolated from HCC cells and patient tissues using TRIzol reagent (Invitrogen). cDNA was synthesized from total RNA using the ImProm-II™ Reverse Transcriptase system (Promega) according to the manufacturer’s protocol and a previous study.^56,57^ The mRNA levels were measured by quantitative real-time PCR (RT-qPCR), which was performed in an ABI 7500 Real-time PCR system using SYBR Green PCR Master Mix (Applied Biosystems). The qPCR primers used are as follows: 5′-CCCGAAGAAGACGTAGAAGATGAC-3′ and 5′-CCCGAAGAAGAGGTGGAGGACGAC-3′ (Tbx3), 5′-CCACCAAAGTCACGCTGAA-3′ and 5′–TGCTTGGATTCCAGAAACG-3′ (E-cadherin), 5′-CGGAACAAGGAGAAGAG CAA-3′ and 5′- GCTCAGCCACCTTCTGTTTTA-3′ (HDAC5), and 5′-TCCATGACAAC TTTGGTATCG-3′ and 5′-TGTAGCCAAATTCGTTGTCA-3′ (GAPDH). Serial 1 to 10 dilutions of plasmid DNA (pIRES-Tbx3, pcDNA-E-cadherin, pCRII-GAPDH) were used as standards for the absolute quantification of each cDNA. The amount of target gene expression could be calculated from the standard curve, which plotted the cycle threshold (Ct) value and the corresponding value of the amount of input DNA.^58^

### Wound healing assay

Cells were seeded in 12-well plates at a similar density. After incubation for 24 h, a 10-μl pipette tip was applied to generate a straight scratch, which simulated a wound. The cells were rinsed with medium twice to remove any floating cells and were then cultured in medium. Wound healing was observed at 24 h and 72 h, and the scratch area was photographed. Triplicate wells were used for each condition, and each experiment was performed three times.

### Transwell assay

The migration ability of stably transfected cells was evaluated using 48-well Boyden Chambers with a polycarbonate membrane with 10-µM pores (Neuro Probe). For the Matrigel invasion assay, the polycarbonate membrane was precoated with Matrigel (BD Biosciences). For the Transwell assay, the polycarbonate membrane was applied directly. The following procedures were performed in a similar manner. Cells (4 × 10^4^) suspended in 50 μl of DMEM supplemented with 1% FBS were placed in the upper chamber, while DMEM supplemented with 10% FBS was added to the lower chamber. After incubation for 6 h, the cells were fixed and stained with hematoxylin. The cells that had migrated were counted by light microscopy at ×100 magnification. The migration and invasion assays were performed as three independent experiments.

### Tissue samples

The paired tissue samples of primary liver cancer and adjacent non-tumor sites were obtained from 54 HCC patients during surgery prior to any therapeutic intervention. All sample collection procedures were approved by the Clinical Research Ethics Committee of the City University of Hong Kong.

### Immunohistochemistry

Briefly, sections were incubated with anti-Tbx3 antibody (sc-17871, Santa Cruz) at 4 °C overnight. After washing with PBS, the sections were incubated with enzyme-labeled IgG for 1 h at room temperature. After washing with PBS, the chromogen was developed for 5 min and was stopped by washing with PBS. Hematoxylin was used as the counterstain.

### Animal study

HepG2 cells with stable expression of ectopic Tbx3 were grown to confluence and harvested as previously described for intrasplenic injection and were resuspended in serum-free DMEM. The mice were sacrificed before the metastasis analysis.^54,59^ The metastases were enumerated using a dissecting microscope. To estimate the tumor volume, the diameter of each metastatic lesion was measured, and the volume of each tumor was calculated on the assumption that the tumors were spherical.

### Statistical analysis

Values in the figures are expressed as means ± SD. A paired *t*-test was performed for the comparison of two groups for statistical analysis. Spearman’s Rank Correlation analysis was used to determine the strength of the relationship between pairs of variables. *P* values < 0.05 were regarded as significant. All statistical analyses were performed using SPSS 21.0 software.

## Electronic supplementary material

SUPPLEMENTAL MATERIA

## References

[1] Bertuccio P (2017). Global trends and predictions in hepatocellular carcinoma mortality. J. Hepatol..

[2] Chi KN (2016). A phase I dose-escalation study of apatorsen (OGX-427), an antisense inhibitor targeting heat shock protein 27 (Hsp27), in patients with castration-resistant prostate cancer and other advanced cancers. Ann. Oncol..

[3] Azevedo CM (2013). Multidimensional optimization of promising antitumor xanthone derivatives. Bioorg. Med. Chem..

[4] He QY, Chen J, Kung HF, Yuen AP, Chiu JF (2004). Identification of tumor-associated proteins in oral tongue squamous cell carcinoma by proteomics. Proteomics.

[5] Torre LA (2015). Global cancer statistics, 2012. CA Cancer J. Clin..

[6] Zhang JF (2011). Primate-specific microRNA-637 inhibits tumorigenesis in hepatocellular carcinoma by disrupting signal transducer and activator of transcription 3 signaling. Hepatology.

[7] Shen Z (2008). The kringle 1 domain of hepatocyte growth factor has antiangiogenic and antitumor cell effects on hepatocellular carcinoma. Cancer Res..

[8] Naiche LA, Harrelson Z, Kelly RG, Papaioannou VE (2005). T-box genes in vertebrate development. Annu. Rev. Genet..

[9] Bamshad M (1997). Mutations in human TBX3 alter limb, apocrine and genital development in ulnar-mammary syndrome. Nat. Genet..

[10] Bamshad M (1999). The spectrum of mutations in TBX3: genotype/phenotype relationship in ulnar-mammary syndrome. Am. J. Hum. Genet..

[11] Linden H, Williams R, King J, Blair E, Kini U (2009). Ulnar mammary syndrome and TBX3: expanding the phenotype. Am. J. Med. Genet. A..

[12] Krajewska M (2005). Analysis of apoptosis protein expression in early-stage colorectal cancer suggests opportunities for new prognostic biomarkers. Clin. Cancer Res..

[13] Lin L (2007). Beta-catenin directly regulates Islet1 expression in cardiovascular progenitors and is required for multiple aspects of cardiogenesis. Proc. Natl Acad. Sci. USA.

[14] Hoogaars WM (2007). Tbx3 controls the sinoatrial node gene program and imposes pacemaker function on the atria. Genes Dev..

[15] Bakker ML (2008). Transcription factor Tbx3 is required for the specification of the atrioventricular conduction system. Circ. Res..

[16] Mesbah K, Harrelson Z, Theveniau-Ruissy M, Papaioannou VE, Kelly RG (2008). Tbx3 is required for outflow tract development. Circ. Res..

[17] Wong K, Peng Y, Kung HF, He ML (2002). Retina dorsal/ventral patterning by Xenopus TBX3. Biochem. Biophys. Res. Commun..

[18] Behesti H, Holt JK, Sowden JC (2006). The level of BMP4 signaling is critical for the regulation of distinct T-box gene expression domains and growth along the dorso-ventral axis of the optic cup. BMC Dev. Biol..

[19] He M, Wen L, Campbell CE, Wu JY, Rao Y (1999). Transcription repression by Xenopus ET and its human ortholog TBX3, a gene involved in ulnar-mammary syndrome. Proc. Natl Acad. Sci. USA.

[20] Coll M, Seidman JG, Muller CW (2002). Structure of the DNA-bound T-box domain of human TBX3, a transcription factor responsible for ulnar-mammary syndrome. Structure.

[21] Carlson H, Ota S, Campbell CE, Hurlin PJ (2001). A dominant repression domain in Tbx3 mediates transcriptional repression and cell immortalization: relevance to mutations in Tbx3 that cause ulnar-mammary syndrome. Hum. Mol. Genet.

[22] Amir S (2016). Regulation of the T-box transcription factor Tbx3 by the tumour suppressor microRNA-206 in breast cancer. Br. J. Cancer.

[23] Arendt LM (2014). Human breast progenitor cell numbers are regulated by WNT and TBX3. PLoS ONE.

[24] Abrahams A, Mowla S, Parker MI, Goding CR, Prince S (2008). UV-mediated regulation of the anti-senescence factor Tbx2. J. Biol. Chem..

[25] Burgucu D (2012). Tbx3 represses PTEN and is over-expressed in head and neck squamous cell carcinoma. BMC Cancer.

[26] Han J (2010). Tbx3 improves the germ-line competency of induced pluripotent stem cells. Nature.

[27] Galan-Caridad JM (2007). Zfx controls the self-renewal of embryonic and hematopoietic stem cells. Cell.

[28] Demay F (2007). T-box factors: targeting to chromatin and interaction with the histone H3 N-terminal tail. Pigment Cell Res..

[29] Miao ZF (2016). Tbx3 overexpression in human gastric cancer is correlated with advanced tumor stage and nodal status and promotes cancer cell growth and invasion. Virchows Arch..

[30] Krstic M (2016). The transcriptional regulator TBX3 promotes progression from non-invasive to invasive breast cancer. BMC Cancer.

[31] Lyng H (2006). Gene expressions and copy numbers associated with metastatic phenotypes of uterine cervical cancer. BMC Genom..

[32] Mlotshwa S (2005). Ectopic DICER-LIKE1 expression in P1/HC-Pro Arabidopsis rescues phenotypic anomalies but not defects in microRNA and silencing pathways. Plant Cell.

[33] Peres J, Mowla S, Prince S (2015). The T-box transcription factor, TBX3, is a key substrate of AKT3 in melanomagenesis. Oncotarget.

[34] Peres J, Prince S (2013). The T-box transcription factor, TBX3, is sufficient to promote melanoma formation and invasion. Mol. Cancer.

[35] Renard CA (2007). Tbx3 is a downstream target of the Wnt/beta-catenin pathway and a critical mediator of beta-catenin survival functions in liver cancer. Cancer Res..

[36] Wang HC, Meng QC, Shan ZZ, Yuan Z, Huang XY (2015). Overexpression of Tbx3 predicts poor prognosis of patients with resectable pancreatic carcinoma. Asian Pac. J. Cancer Prev..

[37] Shan ZZ (2015). Overexpression of Tbx3 is correlated with Epithelial-Mesenchymal Transition phenotype and predicts poor prognosis of colorectal cancer. Am. J. Cancer Res..

[38] Yarosh W (2008). TBX3 is overexpressed in breast cancer and represses p14 ARF by interacting with histone deacetylases. Cancer Res..

[39] Suzuki A, Sekiya S, Buscher D, Izpisua Belmonte JC, Taniguchi H (2008). Tbx3 controls the fate of hepatic progenitor cells in liver development by suppressing p19ARF expression. Development.

[40] Stilo R (2002). TUCAN/CARDINAL and DRAL participate in a common pathway for modulation of NF-kappaB activation. FEBS Lett..

[41] Rozengurt E, Sinnett-Smith J, Eibl G (2018). Yes-associated protein (YAP) in pancreatic cancer: at the epicenter of a targetable signaling network associated with patient survival. Signal Transduct. Target Ther..

[42] Carlson H, Ota S, Song Y, Chen Y, Hurlin PJ (2002). Tbx3 impinges on the p53 pathway to suppress apoptosis, facilitate cell transformation and block myogenic differentiation. Oncogene.

[43] Rodriguez M, Aladowicz E, Lanfrancone L, Goding CR (2008). Tbx3 represses E-cadherin expression and enhances melanoma invasiveness. Cancer Res..

[44] Jacobs JJ (2000). Senescence bypass screen identifies TBX2, which represses Cdkn2a (p19(ARF)) and is amplified in a subset of human breast cancers. Nat. Genet..

[45] Lu J, Li XP, Dong Q, Kung HF, He ML (2010). TBX2 and TBX3: the special value for anticancer drug targets. Biochim. Biophys. Acta.

[46] Li J, Weinberg MS, Zerbini L, Prince S (2013). The oncogenic TBX3 is a downstream target and mediator of the TGF-beta1 signaling pathway. Mol. Biol. Cell.

[47] Kartikasari AE (2013). The histone demethylase Jmjd3 sequentially associates with the transcription factors Tbx3 and Eomes to drive endoderm differentiation. EMBO J..

[48] Douglas NC, Papaioannou VE (2013). The T-box transcription factors TBX2 and TBX3 in mammary gland development and breast cancer. J. Mammary Gland Biol. Neoplasia..

[49] Dan J (2013). Roles for Tbx3 in regulation of two-cell state and telomere elongation in mouse ES cells. Sci. Rep..

[50] Washkowitz AJ, Gavrilov S, Begum S, Papaioannou VE (2012). Diverse functional networks of Tbx3 in development and disease. Wiley Interdiscip. Rev. Syst. Biol. Med..

[51] He ML, Luo MX, Lin MC, Kung HF (2012). MicroRNAs: potential diagnostic markers and therapeutic targets for EBV-associated nasopharyngeal carcinoma. Biochim. Biophys. Acta.

[52] Ma Y (2009). Glucose-regulated protein 78 is an intracellular antiviral factor against hepatitis B virus. Mol. Cell Proteom..

[53] Dong Q (2018). Hsc70 regulates the IRES activity and serves as an antiviral target of enterovirus A71 infection. Antivir. Res..

[54] Hu JJ (2016). HBx-upregulated lncRNA UCA1 promotes cell growth and tumorigenesis by recruiting EZH2 and repressing p27Kip1/CDK2 signaling. Sci. Rep..

[55] Dong L (2015). Growth suppressor lingerer regulates bantam microRNA to restrict organ size. J. Mol. Cell. Biol..

[56] Dong L, Lin F, Wu W, Huang W, Cai Z (2016). Transcriptional cofactor Mask2 is required for YAP-induced cell growth and migration in bladder cancer cell. J. Cancer.

[57] Dong L, Lin F, Wu W, Liu Y, Huang W (2018). Verteporfin inhibits YAP-induced bladder cancer cell growth and invasion via Hippo signaling pathway. Int. J. Med. Sci..

[58] Lu J (2011). MiR-26a inhibits cell growth and tumorigenesis of nasopharyngeal carcinoma through repression of EZH2. Cancer Res..

[59] Zhao T (2016). The effects of genomic polymorphisms in one-carbon metabolism pathways on survival of gastric cancer patients received fluorouracil-based adjuvant therapy. Sci. Rep..

