# Supplementary material for: Novel HDAC5-interacting motifs of Tbx3 are essential for the suppression of E-cadherin expression and for the promotion of metastasis in hepatocellular carcinoma
Source: Signal Transduct Target Ther. 2018 Aug 24;3:22. doi: 10.1038/s41392-018-0025-6 (PMC6107554; doi:10.1038/s41392-018-0025-6)
Supplement: Supplementary file 1 — SUPPLEMENTAL MATERIA [file 41392_2018_25_MOESM1_ESM.pdf]

Supplementary Material

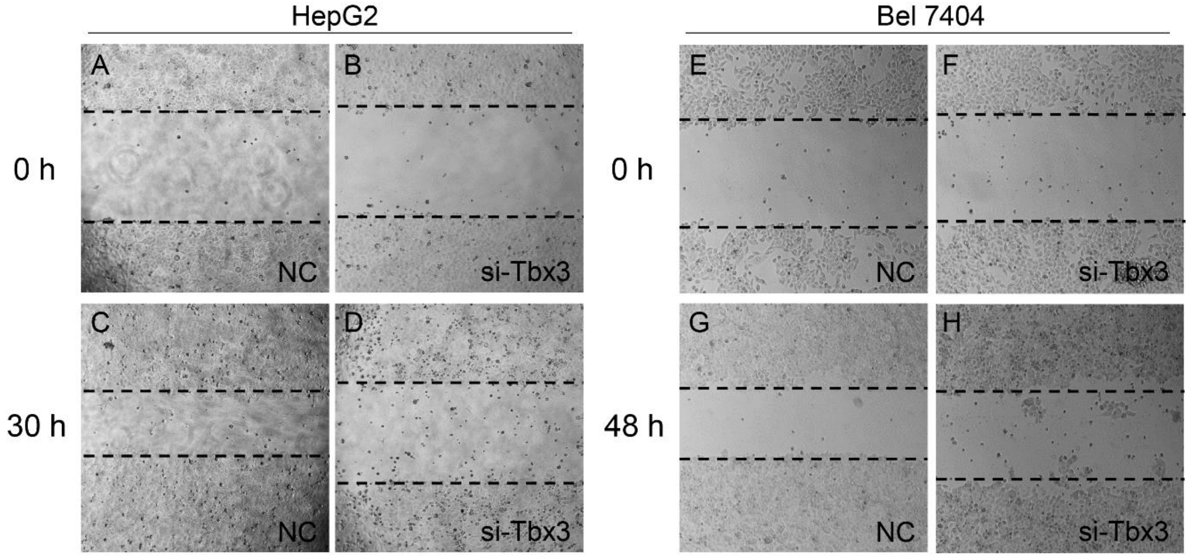

Figure S1. Depletion of Tbx3 represses HCC cell migration.

A-D. Wound healing assay to detect cell migration in HepG2 cells. Decreased migration activity of HepG2 cells displayed after treatment of Tbx3 siRNA; E-H. Wound healing assay to detect cell migration in Bel7404 cells. Downregulated migration activity of Bel7404 cells appears after knockdown of Tbx3 by siRNA treatment.

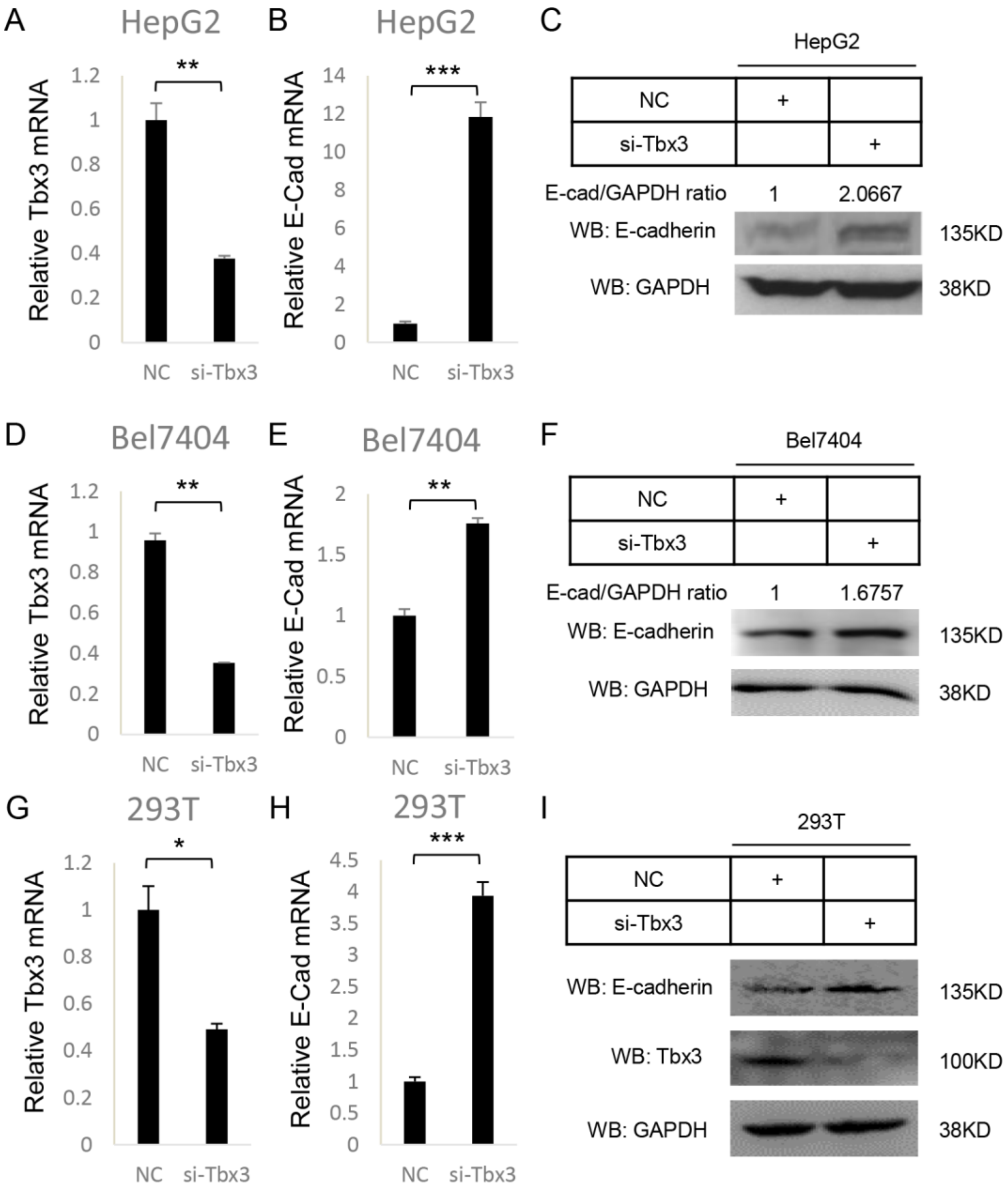

Figure S2. Knockdown of Tbx3 promotes E-cadherin expression.

A, B. RT-qPCR analysis of E-cadherin mRNA level in Tbx3 siRNA treated HepG2 cells; C. WB analysis of E-cadherin in indicating transfected HepG2 cells; D, E. RT-qPCR analysis of E-cadherin mRNA level in Tbx3 siRNA treated Bel7404 cells; F. WB analysis of E-cadherin in indicating transfected Bel7404 cells; G, H. RT-qPCR analysis of E-cadherin mRNA level in Tbx3 siRNA treated 293T cells; I. WB analysis of E-cadherin in indicating transfected 293T cells.

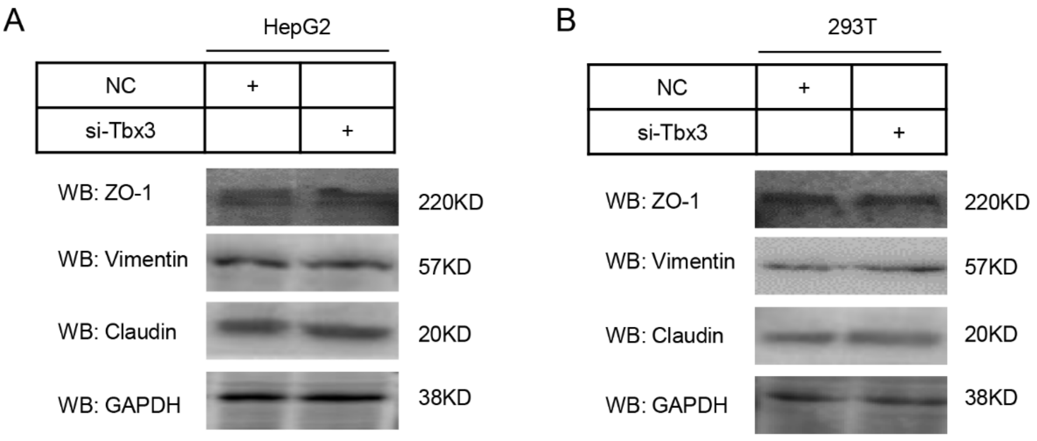

Figure S3. The effect of EMT associated proteins' expression level by si-Tbx3.

A. WB analysis of some EMT related genes' expression (such as ZO-1, Vimentin and Claudin) in indicating transfected HepG2 cells; B. WB analysis of some EMT related genes' expression (such as ZO-1, Vimentin and Claudin) in indicating transfected 293T cells.

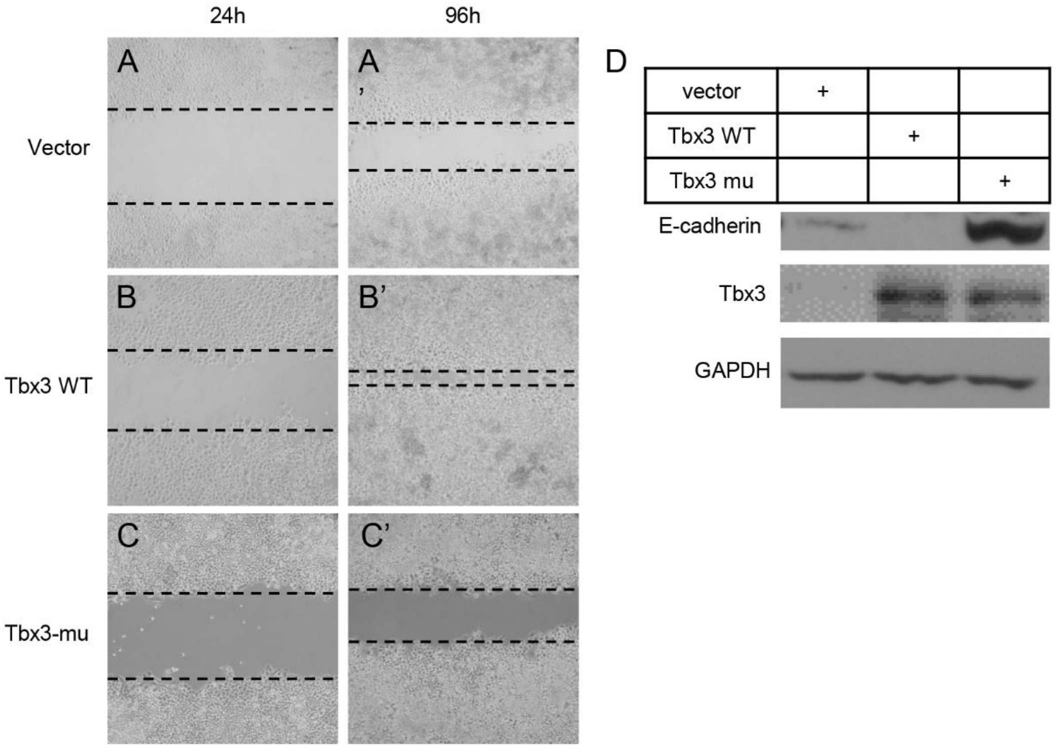

Figure S4. The repression motifs are required for Tbx3 to regulate cell migration and E-cadherin expression in Bel7404 cells.

A-C'. Tbx3 WT, instead of Tbx3 mutant, reduces Bel7404 cell migration activity via wound healing

assays; D. Tbx3 represses E-cadherin expression whereas Tbx3 mutant has no obvious effect in Bel7404 via WB assays.

Table S1. The primers for constructs

Primers	Sequence
G563-9F	5'-AATTCATGGGCTCTTCTGCAGGCGGTGGCGGTGGTGGAGGGCAGGTCTTGGCC-3'
G563-9R	5'- TGCAGAAGAGCCCATGAATTG-3'
G563-77F	5'-TGGCGGTGGTGGAGGGCAGGCTGCCGCAGCAGCGGCTCTGGCAATGTCTC-3'
G563-77R	5'-CCTGCCCTCCACCACCGCCACCGCCTGCAGAAG-3'
G571-577F	5'-TTCCATCTGCAACAGCAGGGCGGAGGCGGAGGCGGTGGGCAATGTCTC-3'
G571-577R	5'-CCTGCTGTTGCAGATGGAAAGGCAGTGCAGA-3'
G579-84F	5'-GGCCTCACAGGGTCTGGCAGGGGGTGGCGGCGCAGCGCTGTTCTCCT-3'
G579-84R	5'-TGCCAGACCCTGTGAGGCCAAGACCTGCTGTTG-3'
G585-91F	5'-TGTCTCCCTTCGGAGGGGGCGGCGGAGGCGGTGGTGGATACATGGCCGC-3'
M585-91R	5'-CCCTCCGAAGGGAGACATTGCCAGACCCTGTGA-3'
G588-94F	5'-CTTCGGAGGGCTGTTCTCCGGCGGTGGTGGAGGCGGAGGCGCGGCTGCT-3'
G588-94R	5'-GGAGAACAGCCCTCCGAAGGGAGACATTGCCAG-3'
G593-99F	5'-TCTCCTACCCTTATACATACGGAGGCGGGGGTGGTGGAGGCTCCTCTGCT-3'
G593-99R	5'-GTATGTATAAGGGTAGGAGAACAGCCCTCCGAA-3'
G604-6F	5' -GCAGCCTCCTCTGCTGTGGGAGGCGGTCCTTTTCTT-3'
G604-6R	5'- CACAGCAGAGGAGGCTGCAGCAGCCGC-3'
D604-6F	5'-GCAGCCTCCTCTGCTGTGGATGATGATCCTTTTCTT-3'
D604-6R	5'-CACAGCAGAGGAGGCTGCAGCAGCCGC-3'
D605F	5'- GCAGCCTCCTCTGCTGTGCACGATCACCCTTTTCTT-3'
A605F	5' -GCAGCCTCCTCTGCTGTGCACGCACACCCTTTTCTT-3'
D/A605R	5'-GTGCACAGCAGAGGAGGCTGCAGCAGCCGC-3'
M613-9F	5'-CTTTTCTTAACGCCGTGGGCGGCGGGGGCGGAGGCGGCCCGTACTCATT-3'
M613-9R	5'-CACGGCGTTAAGAAAAGGGTGTCTGTGCACA-3'
DEL585-99F	5'-TCCTCTGCTGTGCACAGACACCCTTTTCTTAAC-3'
DEL585-99R	5'-CCCTCCGAAGGGAGACATTGCCAGACCCTG-3'
G(585-91)D(604-6)F	5'-CGGGATCCGGCGGCGGAGGCGGT-3'
G(585-91)D(604-6)R	5'-CCAAGCTTTTAATCATCATCCACAGCAGA -3'
